# Integration and scaling of UV‐B radiation effects on plants: from molecular interactions to whole plant responses

**DOI:** 10.1002/ece3.2064

**Published:** 2016-06-21

**Authors:** Vasile Alexandru Suchar, Ronald Robberecht

**Affiliations:** ^1^ Department of Statistical Science College of Science University of Idaho 875 Perimeter Drive MS1104 Moscow Idaho 83844‐1104; ^2^ Department of Forest, Rangeland, and Fire Sciences College of Natural Resources University of Idaho 875 Perimeter Drive MS1133 Moscow Idaho 83844‐1133

**Keywords:** Ecological integration, plant growth, plant modeling, UV‐B radiation

## Abstract

A process based model integrating the effects of UV‐B radiation to molecular level processes and their consequences to whole plant growth and development was developed from key parameters in the published literature. Model simulations showed that UV‐B radiation induced changes in plant metabolic and/or photosynthesis rates can result in plant growth inhibitions. The costs of effective epidermal UV‐B radiation absorptive compounds did not result in any significant changes in plant growth, but any associated metabolic costs effectively reduced the potential plant biomass. The model showed significant interactions between UV‐B radiation effects and temperature and any factor leading to inhibition of photosynthetic production or plant growth during the midday, but the effects were not cumulative for all factors. Vegetative growth were significantly delayed in species that do not exhibit reproductive cycles during a growing season, but vegetative growth and reproductive yield in species completing their life cycle in one growing season did not appear to be delayed more than 2–5 days, probably within the natural variability of the life cycles for many species. This is the first model to integrate the effects of increased UV‐B radiation through molecular level processes and their consequences to whole plant growth and development.

## Introduction

Integration among various ecological processes and scaling among various levels of organization are inherent in ecology and pose major challenges in understanding the consequences of global environmental problems (Levin 1992). Although research on integrating ecological levels has been done (Clark 1990), many ecological studies are still short‐term and small‐scale experiments. Such experiments have limited ecological relevance as more factors are added and the scale is increased (Carpenter 1996; Schindler 1998), and fail in testing the major theories about the natural world (Weiner 1995). Our approach modeled published molecular interactions and the relevant mechanisms responsible for the whole plant responses to ambient and enhanced UV‐B radiation (280–320 nm).

While UV radiation has been a natural environmental stress factor for organisms since the pre‐Cambrian era (Sagan 1973; Lowry et al. 1980; Rettberg et al. 1998; Cockell and Horneck 2001), it may also have contributed partly to the diversity of plant species (Lowry et al. 1980; Stafford 1991; Rozema 1999), which led to feedback effects on atmospheric, terrestrial, and aquatic systems (Kenrick and Crane 1997). Furthermore, at low levels, UV radiation may mediate plant acclimation, and influence growth and development (Rizzini et al. 2011; Fasano et al. 2014). Technical difficulties limited experimental research mostly to individual and subindividual plant levels (DeLucia et al. 2001), and cannot test how the potential UV‐B induced changes may be amplified across higher ecological scales and trophic levels (Caldwell et al. 1998; Warren et al. 2002), or the potential interactions between stratospheric ozone depletion and global warming (Hartman et al. 2000; United Nations Environment Programme, 2012).

Although there is considerable research regarding the effects of UV‐B radiation on subindividual and individual plant levels, we used a modeling approach to integrate these processes and to examine how changes in molecular and cellular processes are scaled to effects at the whole plant level. We modeled the function of an individual plant by integrating photosynthetic production, respiration, and resource allocation. We examined a variety of questions that would be difficult to approach through experimental research, including: (1) What are the most advantageous strategies for the plant to optimize its growth and potential fitness? (2) Does UV‐B radiation interact with other environmental factors? (3) What is the effect of midday photosynthetic depression?

## Model Framework

The plant can be viewed as a system that dynamically balances the resource uptake and use. Plants optimize the resource allocation by investing resources in such a way that maximizes the returns, that is, the growth of organs involved in the acquisition of the limiting resources is promoted (Bloom et al. 1985; Wayne and Bazzaz 1993; Bazzaz 1997; Heilmeier et al. 1997; Cockell 1998). In general, environmental conditions lead to changes in resource allocation and storage, with species growing in variable environments being more plastic in their resource allocation than plants from more stable environments (Chiariello and Gulmon 1991; Miao et al. 1991; Bazzaz 1997; Weiner 2004). This pattern may also apply to a comparison of species with an annual (more plastic) versus a perennial (less plastic) life span. Therefore, the whole plant is the consequence of its life history (Aphalo 2010).

Photosynthetic fixed carbon is synthesized in carbohydrate, then exported to the other plant organs or converted in starch for storage for short‐ or long‐term carbohydrate plant needs (Smith 2005). The sink strength of various plant organs regulates the production and allocation of carbohydrates in plants (Cournede et al. 2006; de Reffye et al. 2008; Mathieu et al. 2009). Growth is in part controlled by nitrogen (N) uptake. When nitrogen is not limiting, growth is proportional to the photosynthesis rate. When N becomes limiting, growth rate slows and carbohydrates are accumulated as starch (Fichner et al. 1995; Schulze and Schulze 1995). While whole plant carbon fixation and nutrient uptake rate are influenced by environmental conditions, carbon fixation rates vary in different leaves on the plant, as well as nutrient uptake rates of different root segments (Bazzaz 1997). The correlation between growth and carbon fixation is generally weak, in part because of the variability in the cost in growth due to other resources availability (i.e., type of N source in soil), and in part as a result of variations in resource allocation patterns (Korner et al. 1979; Crabtree and Bazzaz 1993; Bazzaz 1997).

Resource allocation ratios within plant parts changes with ontogeny (Gedroc et al. 1996; Bazzaz 1997; Weiner 2004), but the annual growth rates of leaves, stems, and roots appear to follow similar isometric scale across many seed plant species (Enquist and Niklas 2002; Niklas and Enquist 2002b). These allometric models consider leaves as the only photosynthetic organs, and assumed that biomass allocated to reproductive plants was either negligible or equally drawn from the pools of leaves, stems, and roots (Enquist and Niklas 2002; Niklas and Enquist 2002b). From the plant architecture perspective, plants are composed of repeating structural elements, with identical or similar combination of organs, specific to individual species (Barthelemy and Caraglio 2007; Nygren and Pallardy 2008; de Reffye et al. 2008). These confirm the similar isometric scaling among plant species, at least for aboveground vegetative organs.

In many species, resource allocation toward reproductive parts occurs only after the plant reaches a certain mass, size, or age (Bazzaz and Catovsky 2001). The importance of mass, size, or age as the trigger of the reproductive parts growth, depends on the species. Also, the required size varies with plant age within same plant species (Schmid et al. 1995; Bazzaz 1997). Regardless of the trigger mechanism, reallocation of resources toward reproduction can be complete, gradual, or resource‐availably based (King and Roughgarden 1982a,b; Reekie and Bazzaz 1987; Bazzaz 1997). Moreover, the allocation to reproductive organs can exceed the maturation capacity of plants, and result in abortion of some of the reproductive organs (Lee and Bazzaz 1982, 1986; Marshall and Ellstrand 1988; Bazzaz 1997). Allocation toward secondary metabolites results in resources reallocated from immediate plant growth, but can result in greater benefits in the long run (Gayler et al. 2008). For example, secondary metabolites are the most important leaf constituents that absorb UV‐B radiation and can prevent the bulk of the incident radiation from reaching the cellular DNA, photosystems, and membranes (Robberecht et al. 1980; Koes et al. 1994; Dixon and Paiva 1995; Winkel‐Shirley 2002).

Ultraviolet‐B radiation can interfere with the plant growth and development in several ways. The photoreceptor UVR8 mediates UV‐B photomorphogenic responses involved in synthesis of secondary metabolites, DNA repair and antioxidative defense (Rizzini et al. 2011; Robson et al. 2015) and may be responsible for growth inhibitions (Fasano et al. 2014). Changes in plant growth due to increased UV‐B radiation were associated with stress‐induced morphogenic responses (SIMR), caused by reactive oxygen species (ROS) production and altered phytohormone transport and metabolism (Potters et al. 2007), oxidative stress, auxin metabolism and microtubili organization changes (Robson et al. 2015). Ultraviolet‐B radiation induced DNA lesions (Sancar 1994; Britt 1995, 1996; Taylor et al. 1997), inhibition of cell division (Gonzalez et al. 1998; Rousseaux et al. 2004), and reduced cell expansion (Wargent et al. 2009; Hectors et al. 2010), or both (Hopkins et al. 2002; Hoffman et al. 2003). As a result of accumulation of DNA damage, UV‐B radiation can induce cell cycle arrest, particularly delays in the G1‐to‐S transition of plant cell cycle, to prevent division of cells with damaged DNA (Jiang et al. 2011). These delays in cell division and expansion may result in significant reduction in leaf area (Suchar and Robberecht 2015). Although photosynthetic rates are not well‐correlated to total leaf area (Bazzaz 1997), a reduction in leaf area may result in reduction in the carbohydrate production of the plant. Moreover, plant protection against increased UV‐B radiation requires investment of resources in metabolic processes. For example, increases in UV‐B radiation generally stimulate the species‐specific production of secondary metabolites and results in changes in the quantity and quality of epidermal absorption (Schmelzer et al. 1988; Li et al. 1993; Dixon and Paiva 1995; Winkel‐Shirley 2002). Also, UV‐B radiation DNA lesions are repaired through enzyme‐driven repair mechanisms (Sancar 1994), that might increase the plant metabolic costs.

The UV‐B radiation interference with plant photosynthesis is more complex. Many studies conducted under glasshouse and environmental chamber conditions show that enhanced UV‐B radiation can impair the photosynthesis by affecting the photosystems and phosphorylation reactions, chloroplast structure, and enzyme activity (Allen et al. 1998; Sullivan and Rozema 1999; Zhou et al. 2007). Field studies using modulated field radiation systems that supplement UV‐B radiation proportionally to the ambient UV‐B regiment show that enhanced UV‐B radiation has no significant effects on the photosynthesis (Searles et al. 2001; Bassman et al. 2002; Bassman and Robberecht 2006; Caldwell et al. 2007).

While increases in leaf respiration were observed when plants were subject to increased UV‐B radiation (Ziska et al. 1992), there are very few studies investigating this aspect (Gwynn‐Jones 2001; Bassman et al. 2003). The studies of the effects of increased UV‐B radiation showed increases in respiration rates from 0 to 280% (Gwynn‐Jones 2001; Bassman et al. 2003). The increases in maintenance respiration might be due to increases in resource demands by the plant tissues for protection and repair in both emerging and mature leaves (Gwynn‐Jones 2001). Since the respiration costs are comparable to the growth costs over a growing season in herbaceous plants, variations in those costs can significantly alter the overall plant growth and productivity (Amthor 1984).

Morphological changes such as reduced leaf area, shoot mass, and plant height are frequently associated with enhanced UV‐B radiation (Searles et al. 2001; Caldwell et al. 2003, 2007). Changes in resource allocation and timing of reproduction has been observed (Demchik and Day 1996; Koti et al. 2005, 2007), but it is not definitive that such changes are direct consequences of increased UV‐B radiation or indirect effects caused by diminished carbohydrates production, or changes in nutrient uptake. Also, increased UV‐B radiation can induce increases in leaf thickness (Yamasaki et al. 2007), decreases in leaf thickness (Kakani et al. 2003), or nonsignificant changes in leaf thickness (Kotilainen et al. 2009). While many of the initial studies of the effects of UV‐B radiation on plants reported increases in leaf thickness (Bornman and Vogelmann 1991), analysis of field studies failed to reveal any significant UV‐B radiation induced changes in leaf thickness (Searles et al. 2001; Ballare et al. 2011).

In general, plants exposed to increased UV‐B radiation exhibit elevated levels of secondary metabolites. The construction costs of flavonoids and related phenolic compounds are generally higher than the average for the leaf mass (Poorter and Villar 1997), and therefore, may lower the conversion efficiency of photosynthetic production in leaf biomass down.

Our research modeled these processes for a hypothetical generalized flowering plant with simple, planophyllic, glabrous, green leaves, and integrated the effects of UV‐B radiation on DNA and the consequences on the plant growth, development and reproduction over one growing season. This generalized flowering plant allowed us to model the influence of UV‐B radiation under a variety of scenarios, including variations in growth characteristics and UV‐B irradiance.

## Conceptual Model

**Figure 1:**
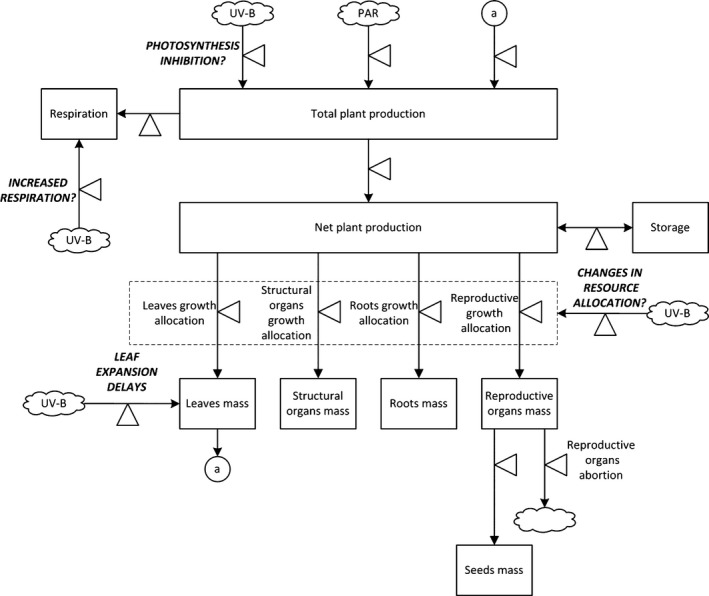
Conceptual model of UV‐B radiation effects on the whole plant.

We chose a process based model to illustrate the effect of UV‐B radiation on the whole plant (Fig. 1). To emphasize the molecular‐to‐whole plant integration under various levels of UV‐B radiation, our model focused on the whole plant function, instead of the plant architecture. Leaf angle can greatly influence the daily effective UV‐B radiation dose intercepted by individual leaves. For example, vertical leaves may receive about 5–41% less daily UV‐B radiation, depending on the latitude and elevation (Caldwell et al. 1980). But it can be also true that some leaves angles will increase the UV‐B radiation interception. Also, since our UV‐B radiation – leaf area model (Suchar and Robberecht 2015) applies to new growth only, it can be assumed that self‐shading is negligible. Total leaf area determines the gross primary production. A fraction of the photosynthetic production is used for respiration, while the remaining production is used toward the growth (Haefner 2005). The remaining photosynthetic production is differentially allocated toward plant organs, following the same proposed isometric rates across the growing season (Enquist and Niklas 2002; Niklas and Enquist 2002a,b). Leaf biomass is correlated with leaf area, leaf area ratio (leaf area per leaf weight) is species‐specific, and respiration rates vary with the total biomass of the plant. Also light interception is proportional with leaf area, and carbon and nitrogen sources and sinks do not interact significantly (i.e., plant growth is not limited by nitrogen uptake). The UV‐B radiation affects whole‐plant growth and development by interfering with leaf expansion, with photosynthesis processes, and respiration (Fig. 1). We considered a generic plant growing over a local growing season. Light interception is proportional with the leaf area and plant leaf architecture effects were considered negligible.

Ultraviolet‐B radiation data were obtained from the UV‐B Monitoring and Research Program (UVMRP) for 10 years 2000–2009, Pullman, Washington, which is a location that is representative of UV‐B radiation for the northern temperate zone. We used UV‐B Langley calibrated data, considered more appropriate than lamp calibrated data for sunny and dry locations (UVMRP 2010). Ultraviolet‐B radiation data were averaged for the 10‐year period, and for each month of the local growing season (May–September). Hourly temperature data was obtained for Spokane, Washington from National Oceanic and Atmospheric Administration – National Climatic Data Center (NOAA 2011). Ambient, 1.5X, 2X unweighted UV‐B radiation levels were simulated.

## Mathematical Model

For the model, the plant was considered to have the following organs: roots (R), aboveground structural organs (S), such as stems, or sheaths and stolons, leaves (L), reproductive organs (Ro), and seeds (Sd).

Since the model considers only the plant function, only the carbon content and its use by different plant pools was considered (Haefner 2005; Kerkhoff et al. 2005). As the plant architecture was not considered, and the modularity of plant structure was not an issue, we modeled the plant growth (i.e., organ appearance) as continuous (Mathieu et al. 2009) and resulting from the source‐sink relationships presented subsequently.

### Total plant production

Under the assumption that leaves are the only photosynthetic organs, total production (P, g time^−1^) is directly proportional with the total leaf mass (Enquist and Niklas 2002; Niklas and Enquist 2002a,b):

(1) $$P\;=\;k_{1}M_{L}$$

Where, *M*_*L*_ is the leaf mass of the plant (g), *k*_1_ is the plant mass photosynthetic production rate multiplier (time^−1^).

Since, generally, the photosynthetic capacity of leaves exhibit a decline after their expansion (Ackerly and Bazzaz 1995; Kitajima et al. 2002), a linear adjustment factor of the decrease of the photosynthetic capacity with time was considered (Kikuzawa 1991; Kitajima et al. 2002). Under the assumption that all leaves in a plant have identical thickness, eq. (1) becomes:

(2) $$P\;=\;k_{1}k_{2}A_{L}\left(1-b_{pd}t\right)$$

Where, *A*_*L*_ is the total leaf area of the plant (m^2^), *k*_2_ is the leaf area ratio (g m^−2^) –*M*_*L*_ = *k*_2_ *A*_*L*_, *b*_*pd*_ is the slope of the linear photosynthetic capacity decline (time^−1^), and *t* is time.

### The total growth of the plant

The total photosynthetic production available for growth (G, g time^−1^) is a function of the total production (P, g time^−1^), the maintenance respiration (R, g time^−1^) and the production allocated to/from storage (S, g time^−1^).

(3) G=P−R±S

### Respiration

It was considered that maintenance respiration (R, g time^−1^) is a function of total plant mass (*M*_*T*_, g).

(4) $$R\;=\;k_{3}M_{T}$$

Where *k*_3_ is the plant mass respiration rate multiplier (time^−1^).

### Plant organ growth

In the basic model, we assumed that all production is allocated to new organ growth from to the common pool of resources.

Under these assumption, the new growth for a new plant organs (G_O_, g time^−1^) becomes

(5) $$G_{O}\;=\;k_{4,O}k_{5,O}G$$

Where, “O” denotes the organ considered (i.e., roots (R), aboveground structural organs (S), leaves (L), reproductive organs (Ro), and seeds (Sd)), *k*_4,*O*_ (unitless) is the conversion efficiency in biomass of photosynthetic production, and *k*_5,*O*_ (unitless) is the percent of total photosynthetic production allocated to the growth of plant organs (Bazzaz 1997; Enquist and Niklas 2002; Niklas and Enquist 2002a,b; Kerkhoff et al. 2005).

For most species, the plant reproduction is associated with a critical plant mass (Geber et al. 1997). However, photoperiod and environmental stress can also initiate flowering in some species (Putterill et al. 2004). Regardless, the minimum mass associated with reproduction can vary with plant age and resource availability (Bazzaz 1997). Since we considered a generalized plant over one growing season, we considered that the plant reproduction is triggered sometime during the growing season, and we simulated different times of beginning of reproduction effect on plant fitness.

For the resource allocation to reproductive parts, we considered a gradual allocation of resources instead complete allocation of resources toward reproductive parts. In this case, the percent of total photosynthetic production allocated toward reproductive parts becomes:

(6) $$k_{5,RO}\;=\;\;\left\{\begin{array}{cc} 0 & if\;t\;<\;t_{br}\\ a_{t}t & if\;t_{br}\;\leq \;t\;<\;\frac{1}{a_{t}}\\ 1 & if\;t\;\geq \;\frac{1}{a_{t}} \end{array}\right.$$

Where, *a*_*t*_ (time^−1^) is the linear increase in photosynthetic production allocation to reproductive parts.

Thus, the proportion of total photosynthetic production allocated toward fruits and seeds follow the same scenario portrayed in eq. (6), and it is limited by the resources available for allocation. The processed is considered to be delayed by ▵*t*_*r*_ (time), the interval necessary for reproduction (i.e., going from flowers to seeds).

### UV‐B radiation effects on whole plant growth and development

The plant model eqs. (1), (2), (4), (5) are adjusted for the effects of UV‐B radiation as follows:

(1.1) $$P\;=\;k_{1,UVB}k_{1}M_{L}$$

(2.1) $$P\;=\;k_{1,UVB}k_{1}k_{2,UVB}k_{2}A_{L}\left(1-b_{pd}t\right)$$

(4.1) $$R\;=\;k_{3,UVB}k_{3}M_{T}$$

(5.1) $$G_{L}\;=\;k_{6,UVB}k_{4,L,UVB}k_{5,L}G$$

Where, *k*_1,*UVB*_ is an adjustment factor due to the effects of UV‐B radiation on photosynthesis, *k*_2,*UVB*_ is an adjustment factor due to effects of UV‐B radiation on leaf thickness, *k*_3,*UVB*_ is an adjustment factor due to the effects of UV‐B radiation on metabolic processes, *k*_4,*L*,*UVB*_ is the conversion efficiency in leaf biomass of photosynthetic production when plant is exposed to increased UV‐B radiation, and *k*_6,*UVB*_ is an leaf growth adjustment factor due to the effects of UV‐B radiation on leaf expansion.

To simulate the UV‐B radiation effects on the leaf area, we used the Suchar and Robberecht (2015) model that simulates relative leaf area for various UV‐B radiation‐induced DNA lesions and rates of photorepair and dark repair.

The variables of interest in our model were UV‐B radiation‐induced relative changes in organ biomass: *M*_*O*,*UVB*_/*M*_*O*_ (“O” denotes the organ considered (i.e., roots (R), stems (S), leaves (L), reproductive organs (Ro), and seeds (Sd)) for the scenarios considered.

## Parameter Estimation

Since we modeled a hypothetical generalized plant, the parameter estimators considered were means calculated for large arrays of species. Therefore, many of these values were obtained from comprehensive plant traits papers (Poorter and Remkes 1990; Searles et al. 2001; Wright et al. 2004; Poorter et al. 2009; Kattge et al. 2011), but not limited to their results.

### Total plant production

Under the assumption that leave are the only photosynthetic organs, total production (P, g time^−1^) is direct proportional with the total leaf mass (Enquist and Niklas 2002; Niklas and Enquist 2002a,b). See eq. (1).

Under field radiation conditions photosynthesis follows two general patterns: first pattern exhibit an increase in photosynthesis in the morning until it reaches saturation, followed by a decrease in the afternoon; second pattern exhibit a gradual increase in photosynthesis in the morning, followed by a midday depression in photosynthesis rates, and another peak in photosynthesis during the afternoon (Larcher 2003; Xu and Shen 2005). The proposed causes for the midday photosynthetic depression include air and leaf temperature, CO_2_ concentration, air and soil moisture content, decrease in leaf water potential, stomatal closure, increases in respiration, photorespiration and mesophyll resistance, developmental stage, circadian rhythm, photosynthate accumulation, decrease in Rubisco activity and photochemical efficiency, and enhanced abscisic acid production (Larcher 2003; Mc Donald 2003; Xu and Shen 2005; Tay et al. 2007). The second peak in net photosynthesis is usually not as pronounced as the first peak (Xu and Shen 2005). Midday depression might be responsible for decreases in productivity of 30–50% or more (Xu and Shen 2005). We simulated two theoretical scenarios: one peak in net photosynthesis, and two‐peak photosynthesis. The maximum net photosynthesis values ranges from 0.008 to 0.14 h^−1^ for herbaceous plant species, and from 0.003 to 0.03 h^−1^ for woody species (Larcher 2003). For our model we considered a mid‐value from the interval of maximum net photosynthesis range which led to a maximum value for *k*_1_ = 0.1 plus the maintenance respiration (Larcher 2003). To account for daily changes in photosynthesis, we considered a generic trend, as follow:

(7) $$k_{1}\;=\;aTime\;+\;bTime^{2}$$

Where time denotes the time step, and ranges from sunrise until sunset (adjusted for time of the year), and a and b coefficients were calculated for the maximum value for *k*_1_ considered, and the time range. Equation coefficients were adjusted for each month of the growing season considered.

For the second trend, plant species exhibiting midday depression, we considered a reduction in photosynthesis around the midday resulting in an average daily reduction in photosynthesis of 40% (Xu and Shen 2005). More specific relationships can be readily substituted for the species of interest. It is noteworthy that increased UV‐B radiation may reduce stomatal conductance, leading to decreased plant water‐loss rates (Nogues et al. 1998). Potentially, it may prevent the development of midday depression, and reduce the severity of drought stress. The interaction of UV‐B radiation and drought stress was not analyzed in the current simulations.

For 45,733 entries, the average specific leaf area was calculated to be 0.0166 m^−2^ g (Kattge et al. 2011), which leads to a value for the leaf area ratio *k*_2_ of 60.24 g m^−2^.

The leaf photosynthetic capacity decline rate seems to be positively correlated with leaf lifespan (Ackerly and Bazzaz 1995; Kitajima et al. 2002). For leaves with longer lifespan (>170 days) as those considered in our model, we considered a loss in photosynthetic capacity of approximately *b*_*pd*_ = 0.1% day^−1^ or *b*_*pd*_ = 0.004% *h* ^−1^ (Kitajima et al. 2002).

### Respiration

Respiration, the fraction of daily production used in the same time, is sensitive to a series of factors including nutrient content, growth and photosynthesis rates, temperature, and plant organs (Poorter et al. 1991; Atkin and Tjoelker 2003; Loveys et al. 2003; Atkin et al. 2005; Lambers et al. 2005). It has been shown that different plant organs exhibit different respiration rates, and these rates are species specific (Reich et al. 2008). Since we modeled a generalized plant, we assumed that the respiration rates are identical in all plant organs. This assumption might not be realistic, but the temperature environment belowground biomass was not available, and therefore a differentiation between organ respiration rates was not possible. But, these rates can be easily adjusted in case of modeling specific species. For the temperature‐dependence of respiration, we considered a general Q10 value of 2.0 (i.e. respiration doubles per 10°C rise in temperature). While the Q10 respiration value is not constant and is dependent on the temperature range used in its calculations and the temperature‐response curve used (Atkin and Tjoelker 2003; Atkin et al. 2005), it was considered a reasonable approximation since all the other parameter estimators in the model are generalized values, averaged over a wide range of species.

Thus, the plant reaches a maximum relative respiration rate at about 50°C, half of the maximum relative respiration rate at 40°C, and negligible respiration at 0°C. At 20°C, the maintenance respiration rates at the beginning of the night range from 0.001 to 0.008 g g^−1^ DM h^−1^ in deciduous species (Larcher 2003). Also, during the night respiration rates continuously decrease by 40–50% until the sunrise (Larcher 2003). A mid‐value was considered. Thus, *k*_3_ equals 0.0045 h^−1^ during the day and the beginning of the night, and reaches 0.0025 h^−1^ at daylight, with the linear night decline in maintenance respiration.

### Plant organ growth

The allometric relationships proposed for a broad range of plant species (Enquist and Niklas 2002; Niklas and Enquist 2002a,b) suggest biomass allocation ratio for Leaves (L): Roots (R): Stems (S) of approximately 0.3:0.13:0.57. By combining these values with the values for the conversion efficiency *k*_4,*O*_ of 0.67:0.75:0.69 (L:R:S) (Poorter and Villar 1997), the estimates for the percent of total photosynthetic production allocated to the growth of plant organs *k*_5,*O*_ = 0.31: 0.12: 0.57 (L:R:S). For a wide range of species, the conversion efficiencies *k*_4,*O*_ to reproductive organs and seeds are 0.71 and 0.65 (Poorter and Villar 1997).

We considered that the duration of flowering is about 1–2 weeks, and the fruit growth and seed maturation is about 1 month. As a result for a May to September growing season considered, the time of beginning of reproduction *t*_*br*_ should be at the latest the end of July. These result in values for *t*_*br*_ = 2160 *h* ^−1^ and for ▵*t*_*r*_ = 168–336 *h* ^−1^.

If we consider a gradual allocation of resources toward reproductive parts of about 2–4 weeks, the value for the linear increase in photosynthetic production allocation to reproductive parts become approximately *a*_*t*_ = 0.0004 *h* ^−1^.

### UV‐B radiation effects on whole plant growth and development

Since field studies with modulated field UV‐B radiation systems indicated that enhanced UV‐B radiation has no significant effects on the photosynthesis (Searles et al. 2001; Bassman et al. 2002; Bassman and Robberecht 2006; Caldwell et al. 2007), we considered in the model that enhanced UV‐B radiation has no significant effects on photosynthetic rate.

Increased UV‐B radiation can result in increases (Yamasaki et al. 2007), decreases (Kakani et al. 2003), or nonsignificant changes in leaf thickness. (Searles et al. 2001; Kotilainen et al. 2009; Ballare et al. 2011). A generic range for the adjustment factor due to effects of UV‐B radiation on leaf thickness, *k*_2,*UVB*_ between 0.75 and 1.25, was considered for the model calibration and validation.

The studies of the effects of increased UV‐B radiation showed increases in respiration rates from 0 to 280% (Gwynn‐Jones 2001; Bassman et al. 2003). Therefore, we considered *k*_3,*UVB*_ a range of 1–4 as the adjustment factor due to the effects of UV‐B radiation on metabolic processes. A final value was inferred from the model calibration procedures.

In general, plants exposed to increased UV‐B radiation exhibit elevated levels of secondary metabolites. The construction costs of flavonoids and related phenolic compounds are generally higher than the average for the leaf mass (Poorter and Villar 1997), and therefore, lowers the conversion efficiency of photosynthetic production in leaf biomass down. For example, an increase in secondary metabolites production by 100% will lower the conversion efficiency from *k*_4,*L*_ = 0.67 to about *k*_4,*L*,*UVB*_ = 0.66 (Poorter and Villar 1997).

For the model of UV‐B radiation effects on the leaf area, we used a Suchar and Robberecht (2015) model that simulates relative leaf area for various UV‐B radiation‐induced DNA lesions and rates of photorepair and dark repair. The model does not include the regulation of plant morphology by UVR8 pathway and SIMR. We recognize that it is a major shortcoming of the model, but it can only be considered when the quantitative relationship between UV‐B radiation dose and photomorphogenic responses is better understood.

## Modeling Methodology

The model was created in Vensim (Systems 2009). Data compilation, preparation, and analysis were done in various programs such as Microsoft Access, Excel, and R‐language.

The models were verified for consistency and units, for correctness of the mathematics and for accuracy of the conceptual logic (Rykiel 1996), calibrated and validated (Shugart 1984; Rykiel 1996; Gardner and Urban 2003). Prior to this, sensitivity analysis procedures were performed (Plentinger and de Penning Vries 1996; Rykiel 1996; Aber et al. 2003).

The variables of interest in our model were UV‐B radiation‐induced relative changes in organ biomass: *M*_*O,UVB*_/*M*_*O*_ (“O” denotes the organ considered (i.e., roots (R), stems (S), leaves (L), reproductive organs (Ro), and seeds (Sd)) for the scenarios considered.

## Model Analysis

### Sensitivity analysis

**Figure 2:**
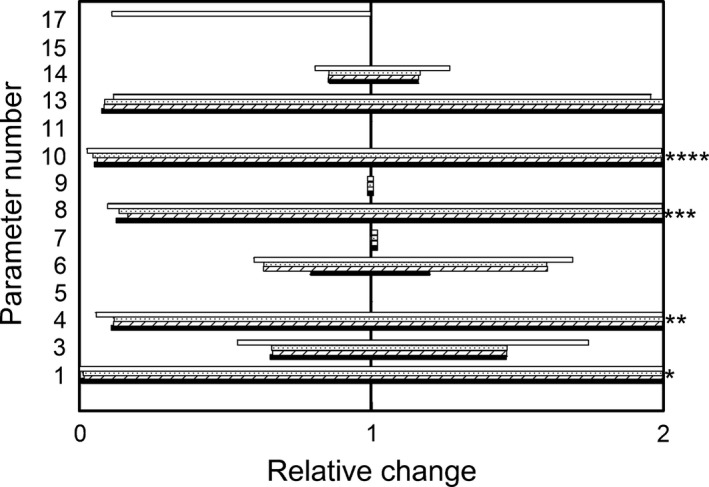
Sensitivity analysis: relative change in roots (solid bar), structural organs (right dash bar), leaves (dotted bar), and mature seeds biomass (no fill bar). Stars indicate relative changes above the scale of the plot.

The parameter values ±25% for the major plant growth model were used in the model sensitivity analysis. For the UV‐B radiation effects on the plant growth and development, the ranges derived for the major model parameters were used for the allowable limits in the model sensitivity analysis. The relative biomass of roots, structural organs, leaves, and mature seeds were measured across the tested model parameters (Fig. 2).

The sensitivity analysis of the model showed that all model output variables considered were highly sensitive to the net production available to growth (production per leaf mass, and respiration per plant mass), and the proportion of net production allocated to structural organs and leaves biomass. The measured variables were moderately sensitive to the decline in leaves photosynthetic capacity in time, the proportion of net production allocated to roots, and the speed of reallocation of resources from vegetative biomass toward the reproductive biomass. The relative biomass of roots, structural organs, leaves, and mature seeds were somewhat or not influenced by changes in conversion efficiency of net production for any plant component. Only seed biomass was influenced by changes in the time required for reproduction. Seed biomass was relatively more sensitive than root, shoot, and leaf biomass to changes in decline in leaves photosynthetic capacity with age, and allocation ratio toward roots and reproductive organs.

The relative biomass of roots, structural organs, leaves, and mature seeds were highly sensitive to UV‐B radiation induced changes in photosynthetic production and metabolism, but not very sensitive to increases in conversion efficiency to leaf biomass due to supplemental metabolic investment in secondary metabolites. The effects of UV‐B radiation on leaf expansion were previously analyzed in Suchar and Robberecht (2015).

Overall the model is highly sensitive to the variation in parameters. If species‐specific data were used instead, the sensitivity analysis would have been conducted for combinations of parameters, and smaller deltas. But since the model was parameterized with averaged data over multiple species, farther sensitivity analysis at this point was considered unnecessary.

### Calibration and validation

Results from meta‐analysis studies of the effects of UV‐B radiation on plant characteristics were used in the calibration and validation process (Searles et al. 2001; Li et al. 2010). Note that since this is a model of a hypothetical plant, a true validation (i.e., testing the model against data unused in the construction of the model) is not possible. In this context, validation of the model means that the output of the model fall within acceptable ranges proposed by the meta‐analysis studies. For field studies simulating 10–20% ozone reduction and >20% ozone depletion, the average reduction in aboveground vegetative biomass ranged from 6% to 9–15%, the average reduction in shoot biomass ranged from 6% to 16%, the average reduction in leaf area ranged from 1.4% to 16.8% (Searles et al. 2001). Nonsignificant changes were recorded for leaf mass per area and reproductive yield (Searles et al. 2001). Similar meta‐analysis recorded for 10–20% ozone reduction and >20% ozone depletion, average reduction in total biomass ranging from 7% to 11.7% for herbaceous plant species, and from nonsignificant to 13.6% in woody plant species, average reduction in leaf area ranging from nonsignificant to 16.1% and 16.8% in herbaceous and wood plant species respectively (Li et al. 2010). The changes in root: shoot ratios were nonsignificant for both ozone depletion categories (Li et al. 2010). The final values considered for our calibration and validation of our model, for conditions simulating 10–20% ozone reduction and >20% ozone depletion, were the following: for average decreases in aboveground biomass 6% to 12.5%; for average decreases in leaf area 1.4% to 16.5%; for shoot biomass 6% to 16%; for reproductive yield 0% for both ozone depletion regimes. Since these averages had 95% confidence intervals of up to ±100%, we considered that if our generic model yields values within the same order of magnitude with those considered for calibration, the model is satisfactory. If it yields values outside these constrains, the model requires further refinement.

The model was calibrated by an iterative process to adjust the most sensitive parameters. The calibration process suggests that enhanced UV‐B radiation may cause increases in the plant metabolic rates, but may be species specific as suggested in literature (Gwynn‐Jones 2001; Bassman et al. 2003). Our simulations suggest a 0.5% increase for UV‐B radiation levels corresponding to about 10% ozone depletion, and a 1% increase for UV‐B radiation levels corresponding to about 20% ozone depletion. Our model uses parameter estimators that were averaged over large numbers of species and experimental conditions, and it was expected to not be able to capture with a high degree of precision the effects of UV‐B radiation on respiration rates.

The average decrease in aboveground and structural organs biomass in our simulations for conditions simulating about 10% and 20% ozone depletion were 4% and 11%, below the values suggested by the literature of 6 and 12.5–16% (Searles et al. 2001; Li et al. 2010), but within the confidence limits pre‐established. The underestimation may be due to simulation of single values for about 10% and 20% stratospheric ozone depletion, while the studies considered in the meta‐analysis (Searles et al. 2001; Li et al. 2010) covered ranges of ozone depletion. The leaf area predicted by our model, overestimated the value suggested by the literature (average decrease of about 4%) for conditions simulating about 10% ozone depletion, but underestimated the value suggested by the literature for conditions simulating about 20% ozone depletion. This suggests that some of the linear relationships used in the model are nonlinear, although it is not possible to identify which relationship has to be re‐evaluated at this time, since our model used averaged values.

The meta‐analysis of published studies suggest that these levels of stratospheric ozone depletion lead to nonsignificant changes in the reproductive yield of the species investigated (Searles et al. 2001). In contrast, our model simulations showed average decreases in the number of mature seeds of 5% to 12%, which may result from the fixed reproduction cycle interval used. If plants optimize the resource allocation by investing resources in such a way that maximizes the return (Bloom et al. 1985; Wayne and Bazzaz 1993; Bazzaz 1997; Heilmeier et al. 1997; Cockell 1998), it is likely that the reproduction will not begin at a fixed time in under environmental conditions. A second source of possible uncertainty in the yield of mature seed is related to the relationship between net production demand posed by fertilized flowers ready to “convert” to seeds and the net production available for growth. Since our model considered biomass as the measurable unit, it is not possible to evaluate the amount of net biomass necessary to convert a particular mass of flowers in a particular mass of seeds. Also, the model quantifies reproductive of seeds as a mass of seeds, and does not account for the variation in number of seeds: mass of seeds ratio.

Even though the source data for our model was relatively heterogeneous, our model was capable of addressing the objectives and major questions of our study. The parameter values resulting in the best fit for the models are given in Table 1. Improved model calibration, optimization and testing can be readily done in Vensim (2009) when most of these parameters are estimated for specific species, or more complete experimental data becomes available.

**Table 1:** Summary of the model parameters estimators

Parameter	Definition	Unit	Range	Assigned values^a^
Total mass production				
1 *k*_1_	Plant mass photosynthetic production rate multiplier	Hour^−1^	See eq. (7)	
2 *k*_2_	Leaf area ratio	g m^−2^	60.24	
3 *b*_*pd*_	Slope of the linear photosynthetic capacity decline^b^	% hour^−1^	0.004	
Respiration				
4 *k*_3_	Plant mass respiration rate multiplier	hour^−1^	0.0025–0.0045	
Plant organs growth				
5 *k*_4,*R*_	Conversion efficiency in root biomass of photosynthetic production	Unitless	0.75	
6 *k*_5,*R*_	Percent of total photosynthetic production allocated to roots growth	Unitless	0.12	
7 *k*_4,*S*_	Conversion efficiency in structural organs biomass of photosynthetic production	Unitless	0.69	
8 *k*_5,*S*_	Percent of total photosynthetic production allocated to structural organs growth	Unitless	0.57	
9 *k*_4,*L*_	Conversion efficiency in leaf biomass of photosynthetic production	Unitless	0.67	
10 *k*_5,*L*_	Percent of total photosynthetic production allocated to leaf growth	Unitless	0.31	
11 *k*_4,*RO*_	Conversion efficiency in reproductive organs biomass of photosynthetic production	Unitless	0.71	
12 *k*_5,*RO*_	Percent of total photosynthetic production allocated to reproductive organs growth	Unitless	0–1	
13 *t*_*br*_	Time triggering reproduction	Hour	2160	
14 *a*_*t*_	Linear increase in photosynthetic production allocation to reproductive parts	Hour^−1^	0.0004	
15 *k*_4,*Sd*_	Conversion efficiency in seed biomass of photosynthetic production	Unitless	0.65	
16 *k*_5,*Sd*_	Percent of total photosynthetic production allocated to seed growth	Unitless	0–1	
17 ▵*t*_*r*_	Interval necessary for reproduction	Hour	168–336	
UV‐B radiation effects on whole plant growth and development				
18 *k*_1,*UVB*_	Adjustment factor due to the effects of UV‐B radiation on photosynthesis	Unitless	0.75–1	1
19 *k*_2,*UVB*_	Adjustment factor due to effects of UV‐B radiation on leaf thickness	Unitless	0.75–1.25	1
20 *k*_3,*UVB*_	Adjustment factor due to the effects of UV‐B radiation on metabolic processes	Unitless	1–4	1.0125|1.025
21 *k*_4,*L*,*UVB*_	Conversion efficiency in leaf biomass of photosynthetic production under increased UV‐B radiation	Unitless	0.66	
22 *k*_6,*UVB*_	Adjustment factor due to the effects of UV‐B radiation on leaf expansion	Suchar and Robberecht (2015)		

a where appropriate.

b Leaf senescence coefficients were chosen to model identical trends as leaf growth processes, and timed for the ending of the growing season considered.

## Results

In addition to the simulations used to analyze the model, we considered the following scenarios: (1) increased UV‐B radiation in different periods of the growing season, (2) increased UV‐B radiation in combination with different epidermal absorption spectra and UV‐B radiation induced DNA lesions repair rates, (3) plants growing under three temperature regimes under increased UV‐B radiation, (4) effects of expedited/delayed reproduction on plant growth and reproduction under increased UV‐B radiation, and (5) effects of midday photosynthetic depression in plant growth under increased UV‐B radiation. To investigate these scenarios, the relative changes in maximum roots, structural organs, leaves, and mature seeds biomass under ambient, 1.5X and 2X ambient UV‐B radiation regime were recorded.

The sensitivity analysis indicated that increased UV‐B radiation may decrease net production, resulting from either increased metabolic rates or reduced photosynthetic rates. Decreases in the conversion efficiency in leaf biomass, due to increased production of secondary metabolites, had no significant influence on the vegetative parts and mature seeds biomass. Also, our model showed that increased UV‐B radiation decreased the biomass of mature seeds, which suggested the probability of reproductive timing shifts in plants as a response mechanism.

**Figure 3:**
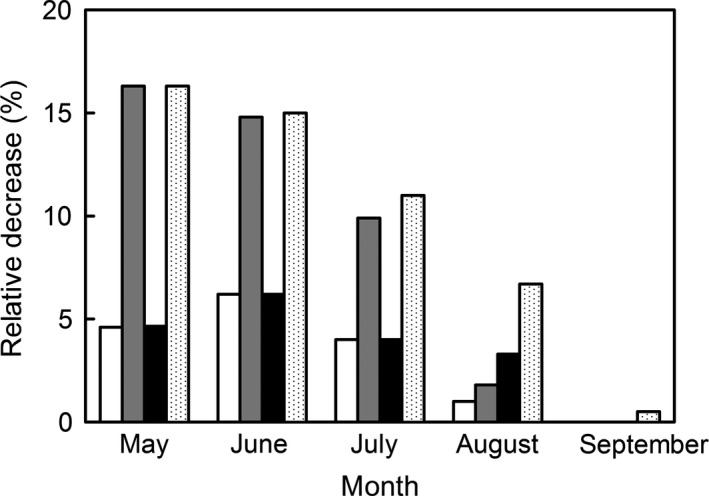
The effect of timing of the increased UV‐B radiation event: relative vegetative and mature seed biomass decrease for plants exposed to increased UV‐B radiation in May, June, July, August, and September (vegetative biomass‐150% UV‐B (no fill bar), vegetative biomass‐200% UV‐B (gray bar), mature seeds biomass‐150% UV‐B (black bar), mature seeds biomass‐200% UV‐B (dotted bar)).

Increased UV‐B radiation in different periods of the growing season simulations showed that plants are more vulnerable to radiation stress in the first part of the growing season, and less sensitive to increase UV‐B radiation in the second part of the growing season (Fig. 3). With fixed timing of reproduction, the biomass of mature seeds was more sensitive than vegetative biomass, and it was disproportionally more affected by increased UV‐B radiation toward the end of the growing season.

**Figure 4:**
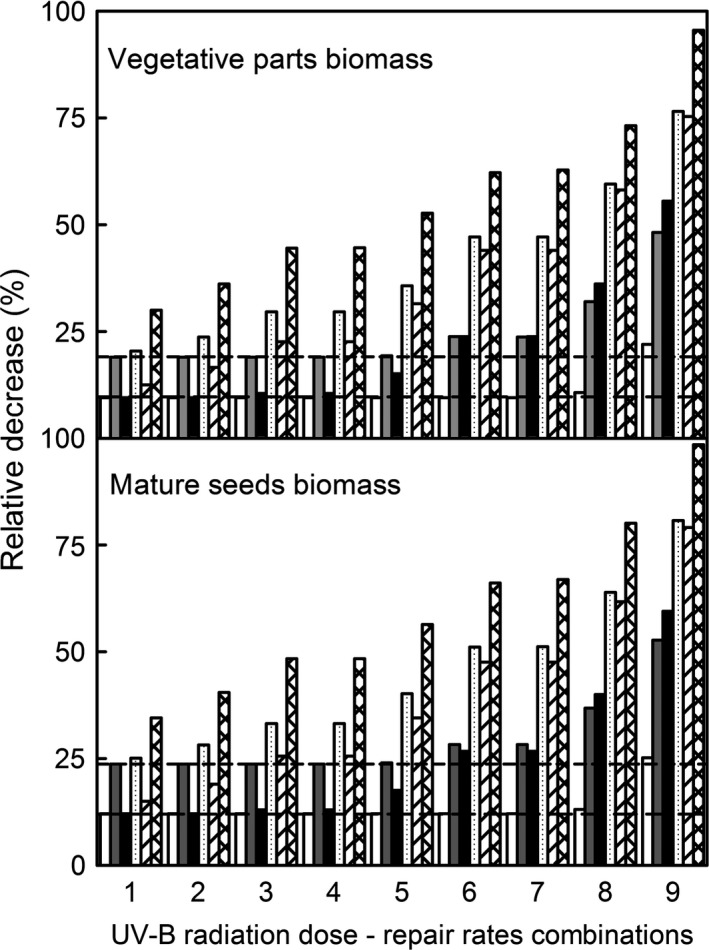
Effect of increased UV‐B radiation in combination with CPD repair rates combinations (1 = high CPD photorepair rate (HP) – high CPD dark repair rate (HD), 2 = HP – average CPD dark repair rate (AP), 3 = HP – low CPD dark repair rate (LD), 4 = average CPD photorepair rate (AP) – HD, 5 = AP – AD, 6 = AP – LD, 7 = low CPD photorepair rate (LP) – HD, 8 = LP – AD, and 9 = LP – LD), relative epidermal absorptance (relative high absorptance at short UV‐B radiation wavelengths: 150% UV‐B (no fill bar) and 200% UV‐B (gray bar), equal absorptance at all UV‐B radiation wavelengths: 150% UV‐B (black bar) and 200% UV‐B (dotted bar), and relative high absorptance at long UV‐B radiation wavelengths: 150% UV‐B (left dash bar) and 200% UV‐B (crisscross bar). Horizontal lines indicate the relative decrease in plant growth due to increased metabolism at 150% UV‐B (long dash line) and 200% UV‐B (medium dash line).

Simulations of increased UV‐B radiation in combination with different epidermal absorption spectra and CPD repair rates showed that increased metabolism was responsible for significant decreases in vegetative biomass and the biomass of mature seeds. The latter was slightly more affected by exposure to UV‐B radiation (Fig. 4). Species with low CPD photorepair and dark repair rates were the most vulnerable. Species with high epidermal UV‐B radiation absorption at short wavelengths exhibited the least growth inhibition even in combination with deficient CPD repair rates, while species with high epidermal UV‐B radiation absorption at long wavelengths were sensitive even when they had high CPD repair rates. Mature seeds biomass showed slightly stronger declines than the whole plant biomass.

**Figure 5:**
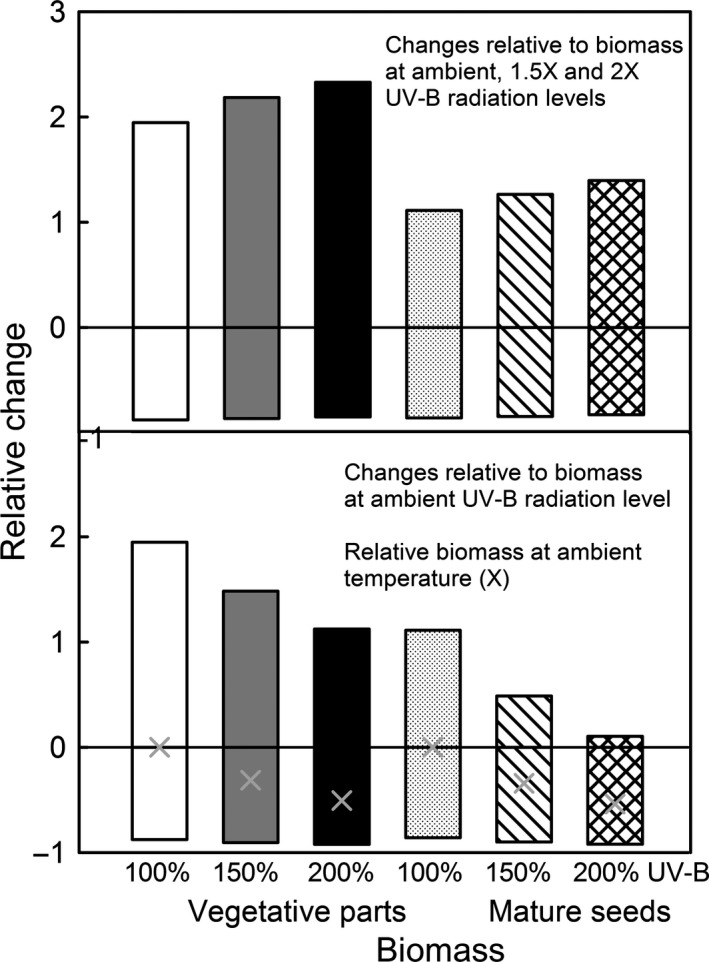
The effect of temperature on growth: relative growth under ambient, −5°C and +5°C temperatures. top: the “0” line represents the plant growth at the ambient temperature in combination with ambient, 1.5X and 2X UV‐B radiation levels. Values above and below the line are relative to these individual values. bottom: the “0” line represents the plant growth at ambient temperature and ambient UV‐B radiation values. Values above and below the line are relative to this unique value.

Simulations of the combined effects of temperature and increased UV‐B radiation, showed that the effect of increased UV‐B radiation effect is confounded with the effects of low temperatures within the range of temperatures considered (Fig. 5). The decrease in vegetative biomass and biomass of mature seeds, for the modeled low temperature range, was similar for the three levels of UV‐B radiation (ambient, 1.5X and 2X ambient). At the higher temperature considered, there was an interaction between temperature and UV‐B radiation. Relative to their growth at ambient temperatures, plants exposed to increased UV‐B radiation exhibited less growth inhibition than plants exposed to ambient UV‐B radiation (Fig. 5 top). Relative to the growth exhibited by plants grown at ambient temperature and UV‐B radiation, plants exposed to increased UV‐B radiation exhibited reduced growth at ambient temperature, but still higher growth at the higher temperatures (Fig. 5 bottom). Mature seeds exhibited similar trends.

**Figure 6:**
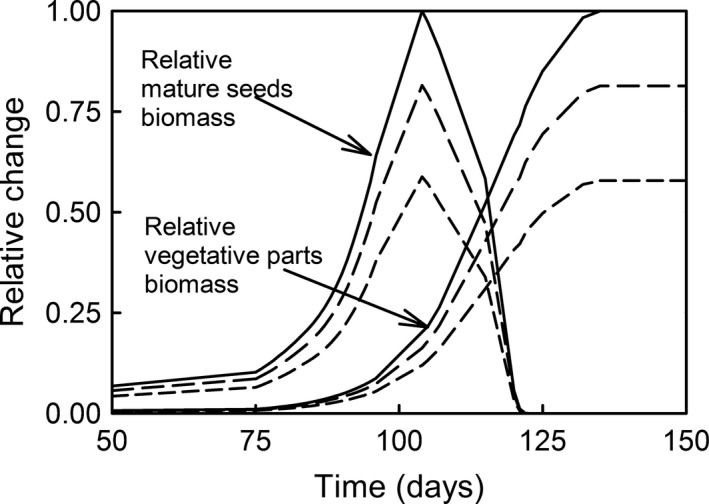
The effect of reproduction timing on maximum vegetative and mature seeds biomass for plants exposed to 100% UV‐B (solid line), 150% UV‐B (long dash line), and 200% UV‐B (medium dash line) radiation.

**Figure 7:**
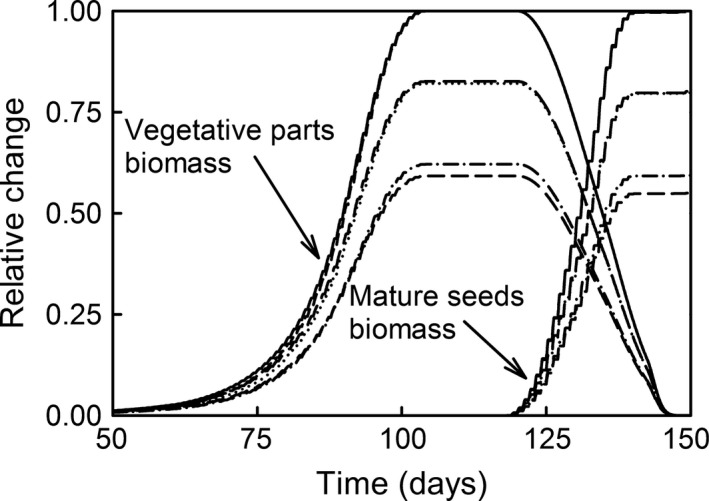
The effect of midday photosynthetic depression on maximum vegetative and mature seeds biomass. Plants without midday depression and exposed to 100% (solid line), 150% (long dash line) and 200% (medium dash line) UV‐B radiation. Plants with midday depression and exposed to 100% (short dash line), 150% (dotted line) and 200% (dot and dash line) UV‐B radiation.

Simulations on reproductive timing under increased UV‐B radiation showed maximum delay in the vegetative biomass of about 5 and 15 days for plants exposed to 150% and 200% UV‐B radiation, respectively (Fig. 6). These delays corresponded with a lack of mature seed production. The delay in vegetative biomass production corresponding to the maximum biomass production for mature seeds ranged from two to five days. Simulations of the effects of midday photosynthetic depression in plant growth under increased UV‐B radiation showed that species exhibiting midday depression were less sensitive to the relatively high doses of UV‐B radiation (Fig. 7). At ambient and 1.5X ambient UV‐B irradiance, species with midday photosynthesis depression exhibited similar growth inhibition, and at 2X ambient UV‐B radiation levels, they exhibited less growth inhibition.

## Discussion

Our simulations suggested that supplemental production of secondary metabolites leads to nonsignificant changes in plant biomass. It was suggested that supplemental investment in secondary metabolites might be a significant drain on the plant resources, and inevitably will affect growth (Johanson et al. 1995; Feldheim and Conner 1996). Our model accounted only for the plant cost in net plant productivity in procuring this extra protection, without considering the potential higher metabolic costs to produce it. It is possible that these additional costs may lead to significant changes in plant biomass due to the production of secondary metabolites. Regardless, the availability of carbohydrates is important in the trade‐off between growth and plant chemical defenses (Gwynn‐Jones 2001), and it has been shown to induce qualitative changes in UV‐B radiation‐induced plant secondary metabolites (Lavola et al. 2003). Also, UV‐B radiation stress‐induced morphological responses may be associated with phytohormone and auxin metabolisms (Potters et al. 2007; Hectors et al. 2012; Robson et al. 2015). Regardless of the cause, our model clearly showed that even small changes in carbohydrate budget of the plant can lead to significant changes in the final plant biomass (Fig. 4). Moreover, species with more efficient and/or higher epidermal absorptance are less susceptible to increased UV‐B radiation (Fig. 4). This confirms previous experimental results that show monocots exhibiting higher sensitivity to increased UV‐B radiation than dicots (Barnes et al. 1990). No level of DNA lesions repair rates can compensate for inefficient UV‐B epidermal absorptance. Since the net production cost of secondary metabolites does not lead to significant decreases in plant biomass, and our simulated supplemental production in secondary metabolites was substantially higher than observed values (Barnes et al. 2016; Siipola et al. 2015), it is plausible to assume that investment in protection to be the most efficient plant response to increased UV‐B radiation. We were unable to identify the potential metabolic costs associated with secondary metabolites production or with other cellular processes, but these aspects may be valuable components of future models.

The inhibition of seed biomass due to increased UV‐B radiation observed in our simulations contradict the results of meta‐analysis studies (Searles et al. 2001; Li et al. 2010) that showed nonsignificant changes in the reproductive yield. This inhibition of seed biomass may be an artifact of fixed reproductive timing in out model simulation comparisons. As shown in Figure 5, the delays in achieving the potential biomass under increased UV‐B radiation are of maximum 5–15 days, and only when seed reproduction is not achieved. If we consider that the plant reproduction may be associated with some critical plant mass (Geber et al. 1997), and that the delays suggested by our model for species that aim to maximize seed production are much smaller (2–5 days) for the growing season considered (probably within the natural variability exhibited within‐species), it is possible that plants response to UV‐B radiation stress may be to delay their reproductive timing, and, thus, maximize their fitness.

The timing of the increased UV‐B irradiance in the environment event seems to be a significant factor (Fig. 3). Plants exposed to increased UV‐B radiation in the beginning of the growing season never recovered to their full biomass potential; while late growing season increased UV‐B events had proportionally smaller effect. These differences are not a direct result of UV‐B radiation (since the actual biomass loss early in the season is smaller than late in the season) but rather a feedback regulation process, were loss of leaf photosynthetic material early in the season, results in higher loss of cumulative primary production. Plant species are more vulnerable to environmental stress during their establishment and initial growth period (Niinemets 2010), and it appears that the effects of UV‐B radiation are also significant during early plant growth and development. Similar results were observed for (*Pisum sativum*) matched pair experiments with combinations of low and high UV‐B radiation levels (Gonzalez et al. 1998). Regardless of the timing of exposure, plants receiving increased UV‐B radiation exhibited reduced vegetative and/or seed biomass. While other environmental stress factors may require morphological and physiological responses to stress conditions (e.g., changes in root: shoot ratio, and/or quantitative/qualitative changes in solute content and concentrations) at the expenses of the vegetative growth, our model suggests that the observed growth inhibitions may be a result of the delay in growth and the timing of the delay, rather than resource availability (note that supplemental production of secondary metabolites do not seem to lead to significant reductions in plant growth and development).

Our model suggests a clear interaction between temperature and UV‐B effect (Fig. 5). For the temperature range considered, plants exhibited similar relative decreases in biomass at lower temperatures for all three levels of UV‐B radiation. Plants exposed to higher temperatures exhibited less relative growth inhibition than plants exposed to ambient UV‐B radiation (Fig. 5 top), and showed higher relative growth at higher temperature than plants exposed to ambient temperature and UV‐B (Fig. 5 bottom).This confirms some experimental results that showed that increases in growing temperature overcompensated for the UV‐B radiation effects in maize and sunflower (Mark and Tevini 1997), but they are different from studies on *Populus tremula* (European Aspen) that showed essentially no increased UV‐B radiation effect at low temperatures, and significant growth inhibitions at higher temperatures (Randriamanana et al. 2015). Since our generalized plant model was parametrized for annuals, it is possible for the simulation results to confirm the observed trends in annual species, and not be similar in all plant growth forms. If correct, our results may suggest that potential increased temperatures due to global change processes might effectively disguise the effects of potential increased UV‐B radiation. The disparity between the effect of UV‐B radiation at high temperatures and low temperatures may be an artifact of the particular low ambient temperatures considered in our model, and characteristic for our region. Simulated low temperatures reduced the photosynthetic production to very little. Therefore, UV‐B radiation‐ induced growth inhibitions were very small proportional with the potential growth. The high temperatures simulated actually increased the photosynthetic production, and the UV‐B induced inhibitions were proportionally higher. This suggests that increases in UV‐B radiation effects may be more visible in highly productive systems, while in low productive plant associations, these effects may be more subtle.

Similar results are suggested by the smaller UV‐B radiation growth inhibition exhibited by species with midday photosynthetic depression (Fig. 7). These results suggest that, generally, any environmental conditions that inhibit photosynthetic production or growth during the midday in particular, and growth in general, will lead to less pronounced UV‐B radiation induced effects. This confirms the results of many studies showing that UV‐B radiation and drought may have confounding effects (Nogués and Baker 2000; Alexieva et al. 2001). Research also indicated that UV‐B radiation and water stress may have synergistic effects (Bjorn et al. 1997), and the addition of UV‐B radiation treatments to drought conditions may have beneficial effects (Balakumar et al. 1993). While we can see how the synergistic effects can emerge from our model under certain combinations of UV‐B and drought simulations, the conditions that might lead to beneficial effects are not fully understood quantitatively and were not included in the model structure. The reason for this merged effect may be due to the nature of UV‐B radiation induced plant growth inhibition. The accumulation of high enough UV‐B radiation‐induced DNA lesions that inhibit plant growth occurs during the midday and early afternoon. If other environmental conditions prevent growth during the same period of the day, the effect of UV‐B radiation cannot be separated. The effects of UV‐B radiation on leaf model used (Suchar and Robberecht 2015) does not include the photomorphogenic responses to UV‐B radiation, which may regulate the gene activity responsible for secondary metabolites production and photorepair of DNA lesions, and may inhibit leaf cell expansion. If these photomorphogenic effects are highly sensitive to the UV‐B radiation dose, and respond readily to changes in the radiation regime, it is possible that the observe effects of daytime environmental driven growth inhibitions and the effects of the UV‐B radiation are confounding. If photomorphogenic responses are less plastic, it is possible that the interaction between the daytime environmental driven growth inhibitions and the effects of the UV‐B radiation are less significant.

Overall, our model suggests that the effects of UV‐B radiation in natural conditions might be less evident as previously thought and may be more in accordance with the results of the latest review studies. Many conditions, such as temperature and humidity can effectively mask the effects of UV‐B radiation. Moreover, while some environmental factors effects can be cumulative with UV‐B radiation effects, other factors might actually prevent the UV‐B radiation to have observable effects to the plant growth (e.g., midday photosynthetic depression and moisture).

We recognize that some of the parameters estimated were derived from unduplicated research, and research that simulated unrealistic conditions. Also, we recognize that the noninclusion of the regulation of plant morphology by UVR8 pathway and SIMR hinders the model predictive power. But this can only be considered when the quantitative relationship between UV‐B radiation dose and photomorphogenic responses is better understood. Moreover, species specific model parameter estimates are necessary. We believe that the direction of the enhanced UV‐B effects are essentially correct, although the presented magnitude of the effects may not be precise. Because of the model framework, we can continue to refine the model as new relevant research becomes available for greater understanding of how UV‐B radiation affects organisms.

## Conclusions

Our model is the first to integrate the effects of increased UV‐B radiation through molecular level processes and their consequences to whole plant growth and development. We modeled the effects of UV‐B radiation at molecular level, and proposed the possible mechanisms that lead to the observed whole plant dynamics. Enhanced UV‐B radiation significantly inhibited plant growth by delaying leaf expansion processes and increasing plant metabolic rates and/or reducing the photosynthesis rate. The costs of effective epidermal UV‐B radiation absorptive compounds did not result in any significant changes in plant growth, but any associated metabolic costs can effectively reduce the potential plant biomass. The model showed significant interactions between UV‐B radiation effects and temperature and any factor leading to inhibition of photosynthetic production or plant growth during the midday, but the effects were not cumulative for all factors. Vegetative growth was significantly delayed in species that do not exhibit reproductive cycles during a growing season, but vegetative growth and reproductive yield in species completing their life cycle in one growing season did not appear to be delayed more than 2–5 days, which is probably within the natural variability of the life cycles for many species. A review of the relevant literature showed a wide range of values for the key parameters. Moreover, certain parameter values were inferred only from the calibration process. However our model allowed the testing of several to examine a variety of questions that were difficult to approach through experimental research.

## Conflict of Interest

None declared.

## References

[ece32064-bib-0001] Aber, J. D. , E. S. Bernhardt , F. A. Dijkstra , R. H. Gardner , K. H. Macneale , W. J. Parton , et al. 2003 Standards of practice for review and publication of models: summary of discussion Pp. 204–210 *in* Canham C. D., Cole J. J., Lauenroth W. K., eds. Models in ecosystem science. Princeton Univ. Press, Princeton, NJ.

[ece32064-bib-0002] Ackerly, D. D. , and F. A. Bazzaz . 1995 Leaf dynamics, self‐shading and carbon gain in seedlings of a tropical pioneer tree. Oecologia 101:289–298. 28307049 10.1007/BF00328814

[ece32064-bib-0003] Alexieva, V. , I. Sergiev , S. Mapelli , and E. Karanov . 2001 The effect of drought and ultraviolet radiation on growth and stress markers in pea and wheat. Plant Cell Environ. 24:1337–1344.

[ece32064-bib-0004] Allen, D. J. , S. Nogues , and N. R. Baker . 1998 Ozone depletion and increased UV‐B radiation: is there a real threat to photosynthesis? J. Exp. Bot. 49:1775–1788.

[ece32064-bib-0005] Amthor, J. S. 1984 The role of maintenance respiration in plant‐growth. Plant Cell Environ. 7:561–569.

[ece32064-bib-0006] Aphalo, P. J. 2010 On how to disentangle the contribution of different organs and processes to the growth of whole plants. J. Exp. Bot. 61:626–628. 20118495 10.1093/jxb/erp398

[ece32064-bib-0007] Atkin, O. K. , and M. G. Tjoelker . 2003 Thermal acclimation and the dynamic response of plant respiration to temperature. Trends Plant Sci. 8:343–351. 12878019 10.1016/S1360-1385(03)00136-5

[ece32064-bib-0008] Atkin, O. K. , D. Bruhn , and M. G. Tjoelker . 2005 Response of plant respiration to changes in temperature: mechanisms and consequences of variations in Q_10_ values and acclimation Pp. 95–135 *in* Lambers H. and Ribas‐Carbo M., eds. Plant respiration: from cell to ecosystem. Springer, The Netherlands.

[ece32064-bib-0010] Balakumar, T. , V. H. B. Vincent , and K. Paliwal . 1993 On the interaction of UV‐B radiation (280–315 nm) with water stress in crop plants. Physiol. Plant. 87:217–222.

[ece32064-bib-0011] Ballare, C. L. , M. M. Caldwell , S. D. Flint , S. A. Robinson , and J. F. Bornman . 2011 Effects of solar ultraviolet radiation on terrestrial ecosystems. Patterns, mechanisms, and interactions with climate change. Photochem. Photobiol. Sci. 10:226–241. 21253661 10.1039/c0pp90035d

[ece32064-bib-0012] Barnes, P. W. , S. D. Flint , and M. M. Caldwell . 1990 Morphological responses of crop and weed species of different growth forms to ultraviolet‐B radiation. Am. J. Bot. 77:1354–1360.

[ece32064-bib-0013] Barnes, P. W. , M. A. Tobler , K. Keefover‐Ring , S. D. Flint , A. E. Barkley , R. J. Ryel , et al. 2016 Rapid modulation of ultraviolet shielding in plants is influenced by solar ultraviolet radiation and linked to alterations in flavonoids. Plant Cell Environ. 39:222–230. 26177782 10.1111/pce.12609

[ece32064-bib-0014] Barthelemy, D. , and Y. Caraglio . 2007 Plant architecture: a dynamic, multilevel and comprehensive approach to plant form, structure and ontogeny. Ann. Bot. 99:375–407. 17218346 10.1093/aob/mcl260 PMC2802949

[ece32064-bib-0015] Bassman, J. H. , and R. Robberecht . 2006 Growth and gas exchange in field‐grown and greenhouse‐grown Quercus rubra following three years of exposure to enhanced UV‐B radiation. Tree Physiol. 26:1153–1163. 16740491 10.1093/treephys/26.9.1153

[ece32064-bib-0016] Bassman, J. H. , G. E. Edwards , and R. Robberecht . 2002 Long‐term exposure to enhanced UV‐B radiation is not detrimental to growth and photosynthesis in Douglas‐fir. New Phytol. 154:107–120.

[ece32064-bib-0017] Bassman, J. H. , G. E. Edwards , and R. Robberecht . 2003 Photosynthesis and growth in seedlings of five forest tree species with contrasting leaf anatomy subjected to supplemental UV‐B radiation. For. Sci. 49:176–187.

[ece32064-bib-0018] Bazzaz, F. A. 1997 Allocation of resources in plants: state of the science and critical questions Pp. 1–37 *in* Bazzaz F. A. and Grace J., eds. Plant resource allocation. Academic Press, San Diego, CA.

[ece32064-bib-0019] Bazzaz, F. A. , and S. Catovsky . 2001 Resource partitioning Pp. 173–184 *in* Simon A. L., ed. Encyclopedia of biodiversity. Elsevier, New York.

[ece32064-bib-0020] Bjorn, L. O. , T. V. Callaghan , I. Johnsen , J. A. Lee , Y. Manetas , N. D. Paul , et al. 1997 The effects of UV‐B radiation on European heathland species. Plant Ecol. 128:252–264.

[ece32064-bib-0021] Bloom, A. J. , F. S. III Chapin , and H. A. Mooney . 1985 Resource limitation in plants ‐ An economic analogy. Annu. Rev. Ecol. Syst. 16:363–392.

[ece32064-bib-0022] Bornman, J. F. , and T. C. Vogelmann . 1991 Effect of UV‐B radiation on leaf optical‐properties measured with fiber optics. J. Exp. Bot. 42:547–554.

[ece32064-bib-0023] Britt, A. B. 1995 Repair of DNA damage induced by ultraviolet radiation. Plant Physiol. 108:891–896. 7630970 10.1104/pp.108.3.891 PMC157437

[ece32064-bib-0024] Britt, A. B. 1996 DNA damage and repair in plants. Annu. Rev. Plant Mol. Biol. 47:75–100. 10.1146/annurev.arplant.47.1.75 15012283

[ece32064-bib-0025] Caldwell, M. M. , R. Robberecht , and W. D. Billings . 1980 A steep latitudinal gradient of solar ultraviolet‐B radiation in the arctic‐alpine life zone. Ecology 61:600–611.

[ece32064-bib-0026] Caldwell, M. M. , L. O. Bjorn , J. F. Bornman , S. D. Flint , G. Kulandaivelu , A. H. Teramura , et al. 1998 Effects of increased solar ultraviolet radiation on terrestrial ecosystems. J. Photochem. Photobiol., B 46:40–52.

[ece32064-bib-0027] Caldwell, M. M. , C. L. Ballare , J. F. Bornman , S. D. Flint , L. O. Bjorn , A. H. Teramura , et al. 2003 Terrestrial ecosystems increased solar ultraviolet radiation and interactions with other climatic change factors. Photochem. Photobiol. Sci. 2:29–38. 12659537 10.1039/b211159b

[ece32064-bib-0028] Caldwell, M. M. , J. F. Bornman , C. L. Ballare , S. D. Flint , and G. Kulandaivelu . 2007 Terrestrial ecosystems, increased solar ultraviolet radiation, and interactions with bother climate change factors. Photochem. Photobiol. Sci. 6:252–266. 17344961 10.1039/b700019g

[ece32064-bib-0029] Carpenter, S. R. 1996 Microcosm experiments have limited relevance for community and ecosystem ecology. Ecology 77:677–680.

[ece32064-bib-0030] Chiariello, N. R. , and S. L. Gulmon . 1991 Stress effects on plant reproduction Pp. 161–188 *in* Mooney H. A., Winter W. E. and Pell E. J., eds. Response of plants to multiple stresses. Academic Press, San Diego.

[ece32064-bib-0031] Clark, J. S. 1990 Integration of ecological levels – individual plant‐growth, population mortality and ecosystem processes. J. Ecol. 78:275–299.

[ece32064-bib-0032] Cockell, C. S. 1998 Biological effects of high ultraviolet radiation on early Earth – a theorethical evaluation. J. Theor. Biol. 193:717–729. 9745762 10.1006/jtbi.1998.0738

[ece32064-bib-0033] Cockell, C. S. , and G. Horneck . 2001 The history of the UV radiation climate of the Earth – theoretical and space‐based observations. Photochem. Photobiol. 73:447–451. 11332042 10.1562/0031-8655(2001)073<0447:thotur>2.0.co;2

[ece32064-bib-0034] Cournede, P. H. , M. Z. Kang , A. Mathieu , J. F. Barczi , H. P. Yan , B. G. Hu , et al. 2006 Structural factorization of plants to compute their functional and architectural growth. Simulation 82:427–438.

[ece32064-bib-0035] Crabtree, R. C. , and F. A. Bazzaz . 1993 Seedling response of 4 birch species to simulated nitrogen deposition – ammonium vs nitrate. Ecol. Appl. 3:315–321. 27759326 10.2307/1941834

[ece32064-bib-0036] DeLucia, E. H. , J. S. Coleman , T. E. Dawson , and R. B. Jackson . 2001 Plant physiological ecology: linking the organism to scales above and below – Ecological Society of America Meeting Snowbird, UT, USA, August 2000. New Phytol. 149:12–16. 10.1046/j.1469-8137.2001.00023-2.x 33853230

[ece32064-bib-0037] Demchik, S. M. , and T. A. Day . 1996 Effect of enhanced UV‐B radiation on pollen quantity, quality, and seed yield in *Brassica rapa* (Brassicaceae). Am. J. Bot. 83:573–579.

[ece32064-bib-0038] Dixon, R. A. , and N. L. Paiva . 1995 Stress‐induced phenylpropanoid metabolism. Plant Cell 7:1085–1097. 12242399 10.1105/tpc.7.7.1085 PMC160915

[ece32064-bib-0039] Enquist, B. J. , and K. J. Niklas . 2002 Global allocation rules for patterns of biomass partitioning in seed plants. Science 295:1517–1520. 11859193 10.1126/science.1066360

[ece32064-bib-0040] Fasano, R. , N. Gonzalez , A. Tosco , F. Dal Piaz , T. Docimo , R. Serrano , et al. 2014 Role of *Arabidopsis* UV resistance locus 8 in plant growth reduction under osmotic stress and low levels of UV‐B. Mol. Plant 7:773–791. 24413416 10.1093/mp/ssu002

[ece32064-bib-0041] Feldheim, K. , and J. K. Conner . 1996 The effects of increased UV‐B radiation on growth, pollination success, and lifetime female fitness in two *Brassica* species. Oecologia 106:284–297. 28307316 10.1007/BF00334556

[ece32064-bib-0042] Fichner, K. , G. W. Koch , and H. A. Mooney . 1995 The photosynthesis‐nitrogen relationship in wild plants Pp. 133–144 *in* Schulze E.‐D. and Caldwell M. M., eds. Ecophophysiology of photosynthesis. Springer‐Verlag, Berlin.

[ece32064-bib-0043] Gardner, R. H. , and D. L. Urban . 2003 Model validation and testing: past lessons, present concerns, future prospects Pp. 184–203 *in* Canham C. D., Cole J. J. and Lauenroth W. K., eds. Models in ecosystem science. Princeton Univ. Press, Princeton, NJ.

[ece32064-bib-0044] Gayler, S. , T. E. E. Grams , W. Heller , D. Treutter , and E. Priesack . 2008 A dynamical model of environmental effects on allocation to carbon‐based secondary compounds in juvenile trees. Ann. Bot.‐Lond. 101:1089–1098. 10.1093/aob/mcm169 PMC2710266 17693454

[ece32064-bib-0045] Geber, M. A. , M. A. Watson , and H. de Kroon . 1997 Organ preformation, development, and resource allocation in perennials Pp. 113–141 *in* Bazzaz F. A. and Grace J., eds. Plant resource allocation. Academic Press, San Diego, CA.

[ece32064-bib-0046] Gedroc, J. J. , K. D. M. McConnaughay , and J. S. Coleman . 1996 Plasticity in root shoot partitioning: optimal, ontogenetic, or both? Funct. Ecol. 10:44–50.

[ece32064-bib-0047] Gonzalez, R. , R. Mepsted , A. R. Wellburn , and N. D. Paul . 1998 Non‐photosynthetic mechanisms of growth reduction in pea (*Pisum sativum* L.) exposed to UV‐B radiation. Plant Cell Environ. 21:23–32.

[ece32064-bib-0048] Gwynn‐Jones, D. 2001 Short‐term impacts of enhanced UV‐B radiation on photo‐assimilate allocation and metabolism: a possible interpretation for time‐dependent inhibition of growth. Plant Ecol. 154:65–73.

[ece32064-bib-0049] Haefner, J. W. 2005 Modeling biological systems: principles and applications, 2nd edn Springer Science+Business Media, New York, NY.

[ece32064-bib-0050] Hartman, D. L. , J. M. Wallace , V. Limpasuvan , D. W. J. Thompson , and J. R. Holton . 2000 Can ozone depletion and global warming interact to produce rapid climate change? Proc. Natl Acad. Sci. USA 97:1412–1417. 10677475 10.1073/pnas.97.4.1412 PMC26447

[ece32064-bib-0051] Hectors, K. , E. Jacques , E. Prinsen , Y. Guisez , J. P. Verbelen , M. A. Jansen , et al. 2010 UV radiation reduces epidermal cell expansion in leaves of *Arabidopsis thaliana* . J. Exp. Bot. 61:4339–4349. 20702567 10.1093/jxb/erq235

[ece32064-bib-0052] Hectors, K. , S. van Oevelen , Y. Guisez , E. Prinsen , and M. A. K. Jansen . 2012 The phytohormone auxin is a component of the regulatory system that controls UV‐mediated accumulation of flavonoids and UV‐induced morphogenesis. Physiol. Plant. 145:594–603. 22304327 10.1111/j.1399-3054.2012.01590.x

[ece32064-bib-0053] Heilmeier, H. , M. Erhard , and E.‐D. Schulze . 1997 Biomass allocation and water use under arid conditions Pp. 93–111 *in* Bazzaz F. A. and Grace J., eds. Plant resource allocation. Academic Press, San Diego, CA.

[ece32064-bib-0054] Hoffman, R. W. , B. D. Campbell , S. J. Bloor , E. E. Swinny , K. R. Markham , K. G. Ryan , et al. 2003 Responses to UV‐B radiation in *Trifolium repens* l. ‐ physiological links to plant productivity and water availability. Plant Cell Environ. 26:603–612.

[ece32064-bib-0055] Hopkins, L. , M. A. Bond , and A. K. Tobin . 2002 Ultraviolet‐B radiation reduces the rates of cell division and elongation in the primary leaf of wheat (*Triticum aestivum* L. cv Maris Huntsman). Plant Cell Environ. 25:617–624.

[ece32064-bib-0056] Jiang, L. , Y. Wang , L. Björn , and S. Li . 2011 UV‐B‐induced DNA damage mediates expression changes of cell cycle regulatory genes in *Arabidopsis* root tips. Planta 233:831–841. 21221633 10.1007/s00425-010-1340-5

[ece32064-bib-0057] Johanson, U. , C. Gehrke , L. O. Bjorn , and T. V. Callaghan . 1995 The effects of enhanced UV‐B radiation on the growth of Dwarf shrubs in a sub‐arctic heathland. Funct. Ecol. 9:713–719.

[ece32064-bib-0058] Kakani, V. G. , K. R. Reddy , D. Zhao , and A. R. Mohammed . 2003 Effects of ultraviolet‐B radiation on cotton (*Gossypium hirsutum* L.) morphology and anatomy. Ann. Bot.‐London 91:817–826. 10.1093/aob/mcg086 PMC4242390 12770842

[ece32064-bib-0059] Kattge, J. , S. Diaz , S. Lavorel , C. Prentice , P. Leadley , G. Bonisch , et al. 2011 TRY ‐ a global database of plant traits. Global Change Biol. 17:2905–2935.

[ece32064-bib-0060] Kenrick, P. , and P. R. Crane . 1997 The origin and early evolution of plants on land. Nature 389:33–39.

[ece32064-bib-0061] Kerkhoff, A. J. , B. J. Enquist , J. J. Elser , and W. F. Fagan . 2005 Plant allometry, stoichiometry and the temperature‐dependence of primary productivity. Global Ecol. Biogeogr. 14:585–598.

[ece32064-bib-0062] Kikuzawa, K. 1991 A cost‐benefit‐analysis of leaf habit and leaf longevity of trees and their geographical pattern. Am. Nat. 138:1250–1263.

[ece32064-bib-0063] King, D. , and J. Roughgarden . 1982a Graded allocation between vegetative and reproductive growth for annual plants in growing seasons of random length. Theor. Popul. Biol. 22:1–16.

[ece32064-bib-0064] King, D. , and J. Roughgarden . 1982b Multiple switches between vegetative and reproductive growth in annual plants. Theor. Popul. Biol. 21:194–204.

[ece32064-bib-0065] Kitajima, K. , S. S. Mulkey , M. Samaniego , and S. Joseph Wright . 2002 Decline of photosynthetic capacity with leaf age and position in two tropical pioneer tree species. Am. J. Bot. 89:1925–1932. 21665621 10.3732/ajb.89.12.1925

[ece32064-bib-0066] Koes, R. E. , F. Quattrocchio , and J. N. M. Mol . 1994 The flavonoid biosynthetic‐pathway in plants – function and evolution. BioEssays 16:123–132.

[ece32064-bib-0067] Korner, C. , J. A. Scheel , and H. Bauer . 1979 Maximum leaf diffusive conductance in vascular plants. Photosynthetica 13:45–82.

[ece32064-bib-0068] Koti, S. , K. R. Reddy , V. R. Reddy , V. G. Kakani , and D. Zhao . 2005 Interactive effects of carbon dioxide, temperature, and ultraviolet‐B radiation on soybean (*Glycine max* L.) flower and pollen morphology, pollen production, germination, and tube lengths. J. Exp. Bot. 56:725–736. 15611147 10.1093/jxb/eri044

[ece32064-bib-0069] Koti, S. , K. R. Reddy , V. G. Kakani , D. Zhao , and W. Gao . 2007 Effects of carbon dioxide, temperature and ultraviolet‐B radiation and their interactions on soybean (*Glycine max* L.) growth and development. Environ. Exp. Bot. 60:1–10.

[ece32064-bib-0070] Kotilainen, T. , T. Venäläinen , R. Tegelberg , A. Lindfors , R. Julkunen‐Tiitto , S. Sutinen , et al. 2009 Assessment of UV biological spectral weighting functions for phenolic metabolites and growth responses in silver birch seedlings. Photochem. Photobiol. 85:1346–1355. 19682323 10.1111/j.1751-1097.2009.00597.x

[ece32064-bib-0071] Lambers, H. , S. A. Robinson , and M. Ribas‐Carbo . 2005 Regulation of respiration in vivo Pp. 1–15 *in* Lambers H. and Ribas‐Carbo M., eds. Plant respiration: from cell to ecosystem. Springer, The Netherlands.

[ece32064-bib-0072] Larcher, W. 2003 Physiological plant ecology, 4th edn Springer‐Verlag, Berlin, 513 pp.

[ece32064-bib-0073] Lavola, A. , P. J. Aphalo , M. Lahti , and R. Julkunen‐Tiitto . 2003 Nutrient availability and the effect of increasing UV‐B radiation on secondary plant compounds in Scots pine. Environ. Exp. Bot. 49:49–60.

[ece32064-bib-0074] Lee, T. D. , and F. A. Bazzaz . 1982 Regulation of fruit maturation pattern in an annual legume, *Cassia‐fasciculata* . Ecology 63:1374–1388.

[ece32064-bib-0075] Lee, T. D. , and F. A. Bazzaz . 1986 Maternal regulation of fecundity – nonrandom ovule abortion in *Cassia‐fasciculata* Michx. Oecologia 68:459–465. 28311795 10.1007/BF01036755

[ece32064-bib-0076] Levin, S. A. 1992 The problem of pattern and scale in ecology. Ecology 73:1943–1967.

[ece32064-bib-0077] Li, J. , T. Ou‐Lee , R. Raba , R. G. Admundson , and R. L. Last . 1993 Arabidopsis flavonoids mutants are hypersensitive to UV‐B irradiation. Plant Cell 5:171–179. 12271060 10.1105/tpc.5.2.171 PMC160260

[ece32064-bib-0078] Li, F.‐R. , S.‐L. Peng , B.‐M. Chen , and Y.‐P. Hou . 2010 A meta‐analysis of the responses of woody and herbaceous plants to elevated ultraviolet‐B radiation. Acta Oecologica 36:1–9.

[ece32064-bib-0079] Loveys, B. R. , L. J. Atkinson , D. J. Sherlock , R. L. Roberts , A. H. Fitter , and O. K. Atkin . 2003 Thermal acclimation of leaf and root respiration: an investigation comparing inherently fast‐ and slow‐growing plant species. Global Change Biol. 9:895–910.

[ece32064-bib-0080] Lowry, B. , D. Lee , and C. Hebant . 1980 The origins of land plants: a new look at an old problem. Taxon 29:183–197.

[ece32064-bib-0081] Mark, U. , and M. Tevini . 1997 Effects of solar ultraviolet‐B radaition, temperature and CO2 on growth and physiology of sunflower and maize seedlings Pp. 224–234 *in* Rozema J., Gieskes W. W. C., van de Geijn S. C., Nolan C. and de Boois H., eds. UV‐B and biosphere. Springer Science, +Business Media, B.V.

[ece32064-bib-0082] Marshall, D. L. , and N. C. Ellstrand . 1988 Effective mate choice in wild radish – evidence for selective seed abortion and its mechanism. Am. Nat. 131:739–756.

[ece32064-bib-0083] Mathieu, A. , P. H. Cournede , V. Letort , D. Barthelemy , and P. de Reffye . 2009 A dynamic model of plant growth with interactions between development and functional mechanisms to study plant structural plasticity related to trophic competition. Ann. Bot. 103:1173–1186. 19297366 10.1093/aob/mcp054 PMC2685317

[ece32064-bib-0084] Mc Donald, M. S. 2003 Photobiology of higher plants. John Wiley & Sons, UK, 354 pp.

[ece32064-bib-0085] Miao, S. L. , F. A. Bazzaz , and R. B. Primack . 1991 Effects of maternal nutrient pulse on reproduction of two colonizing *Plantago* species. Ecology 72:586–596.

[ece32064-bib-0086] Niinemets, U. 2010 Responses of forest trees to single and multiple environmental stresses from seedlings to mature plants: past stress history, stress interactions, tolerance and acclimation. Forest Ecol. Manag. 260:1623–1639.

[ece32064-bib-0087] Niklas, K. J. , and B. J. Enquist . 2002a Canonical rules for plant organ biomass partitioning and annual allocation. Am. J. Bot. 89:812–819. 21665681 10.3732/ajb.89.5.812

[ece32064-bib-0088] Niklas, K. J. , and B. J. Enquist . 2002b On the vegetative biomass partitioning of seed plant leaves, stems, and roots. Am. Nat. 159:482–497. 18707431 10.1086/339459

[ece32064-bib-0542] NOAA (2011) National Climatic Center. http://www.ncdc.noaa.gov/. Accessed May 2011.

[ece32064-bib-0089] Nogués, S. , and N. R. Baker . 2000 Effects of drought on photosynthesis in Mediterranean plants grown under enhanced UV‐B radiation. J. Exp. Bot. 51:1309–1317. 10937707 10.1093/jxb/51.348.1309

[ece32064-bib-0090] Nogues, S. , D. J. Allen , J. I. L. Morison , and N. R. Baker . 1998 Ultraviolet‐B radiation effects on water relations, leaf development, and photosynthesis in droughted pea plants. Plant Physiol. 117:173–181. 9576786 10.1104/pp.117.1.173 PMC35000

[ece32064-bib-0091] Nygren, P. , and S. G. Pallardy . 2008 Applying a universal scaling model to vascular allometry in a single‐Stemmed, monopodially branching deciduous tree (Attim's model). Tree Physiol. 28:1–10. 17938108 10.1093/treephys/28.1.1

[ece32064-bib-0092] Plentinger, M. C. , and de Penning Vries F. W. T. eds. 1996 CAMASE register of agro‐ecosystems models, http://library.wur.nl/way/bestanden/clc/1763788.pdf electronic ed 420 pp.

[ece32064-bib-0093] Poorter, H. , and C. Remkes . 1990 Leaf‐area ratio and net assimilation rate of 24 wild‐species differing in relative growth‐rate. Oecologia 83:553–559. 28313192 10.1007/BF00317209

[ece32064-bib-0094] Poorter, H. , and R. Villar . 1997 Fate of acquired carbon in plants: chemical composition and construction costs Pp. 39–72 *in* Bazzaz F. A. and Grace J., eds. Plant resource allocation. Academic Press, San Diego, CA.

[ece32064-bib-0095] Poorter, H. , A. Vanderwerf , O. K. Atkin , and H. Lambers . 1991 Respiratory energy‐requirements of roots vary with the potential growth‐rate of a plant‐species. Physiol. Plant. 83:469–475.

[ece32064-bib-0096] Poorter, H. , U. Niinemets , L. Poorter , I. J. Wright , and R. Villar . 2009 Causes and consequences of variation in leaf mass per area (LMA): a meta‐analysis. New Phytol. 182:565–588. 19434804 10.1111/j.1469-8137.2009.02830.x

[ece32064-bib-0097] Potters, G. , T. P. Pasternak , Y. Guisez , K. J. Palme , and M. A. K. Jansen . 2007 Stress‐induced morphogenic responses: growing out of trouble? Trends Plant Sci. 12:98–105. 17287141 10.1016/j.tplants.2007.01.004

[ece32064-bib-0098] Putterill, J. , R. Laurie , and R. Macknight . 2004 It's time to flower: the genetic control of flowering time. BioEssays 26:363–373. 15057934 10.1002/bies.20021

[ece32064-bib-0099] Randriamanana, T. R. , A. Lavola , and R. Julkunen‐Tiitto . 2015 Interactive effects of supplemental UV‐B and temperature in European aspen seedlings: implications for growth, leaf traits, phenolic defense and associated organisms. Plant Physiol. Biochem. 93:84–93. 25766888 10.1016/j.plaphy.2015.03.001

[ece32064-bib-0100] Reekie, E. G. , and F. A. Bazzaz . 1987 Reproductive effort in plants. 1. Carbon allocation to reproduction. Am. Nat. 129:876–896.

[ece32064-bib-0101] de Reffye, P. , E. Heuvelink , D. Barthélémy , and deCournè P. H. . 2008 Plant growth models Pp. 2824–2837 *in* Sven Erik J., Brian F., eds. Encyclopedia of ecology. Academic Press, Oxford.

[ece32064-bib-0102] Reich, P. B. , M. G. Tjoelker , K. S. Pregitzer , I. J. Wright , J. Oleksyn , and J. L. Machado . 2008 Scaling of respiration to nitrogen in leaves, stems and roots of higher land plants. Ecol. Lett. 11:793–801. 18445031 10.1111/j.1461-0248.2008.01185.x

[ece32064-bib-0103] Rettberg, P. , G. Horneck , W. Strauch , R. Facius , and G. Seckmeyer . 1998 Simulation of planetary UV radiation climate on the example of the early Earth. Adv. Space Res. 22:335–339.

[ece32064-bib-0104] Rizzini, L. , J. J. Favory , C. Cloix , D. Faggionato , A. O'Hara , E. Kaiserli , et al. 2011 Perception of UV‐B by the *Arabidopsis* UVR8 protein. Science 332:103–106. 21454788 10.1126/science.1200660

[ece32064-bib-0105] Robberecht, R. , M. M. Caldwell , and W. D. Billings . 1980 Leaf ultraviolet optical properties along a latitudinal gradient in the arctic‐alpine life zone. Ecology 61:612–619.

[ece32064-bib-0106] Robson, T. M. , K. Klem , O. Urban , and M. A. K. Jansen . 2015 Re‐interpreting plant morphological responses to UV‐B radiation. Plant Cell Environ. 38:856–866. 24890713 10.1111/pce.12374

[ece32064-bib-0107] Rousseaux, M. C. , S. D. Flint , P. S. Searles , and M. M. Caldwell . 2004 Plant responses to current solar ultraviolet‐B radiation and to supplemented solar ultraviolet‐B radiation simulating ozone depletion: an experimental comparison. Photochem. Photobiol. 80:224–230. 15362944 10.1562/2004-03-30-RA-129

[ece32064-bib-0108] Rozema, J. 1999 UV‐B radiation and terrestrial ecosystems: processes, structure and feedback loops Pp. 101–116 *in* Rozema J., ed. Stratospheric ozone depletion: the effects of enhanced UV‐B radiation on terrestrial ecosystems. Backhuys Publishers, Leiden, The Netherlands.

[ece32064-bib-0109] Rykiel, J. E. J. 1996 Testing ecological models: the meaning of validation. Ecol. Model. 90:229.

[ece32064-bib-0110] Sagan, C. 1973 Ultraviolet selection pressure on earliest organisms. J. Theor. Biol. 39:195–200. 4741712 10.1016/0022-5193(73)90216-6

[ece32064-bib-0111] Sancar, A. 1994 Structure and function of DNA photolyase. Biochemistry 33:2–9. 8286340 10.1021/bi00167a001

[ece32064-bib-0112] Schindler, D. W. 1998 Whole‐ecosystem experiments: replication versus realism: the need for ecosystem‐scale experiments. Ecosystems 1:323–334.

[ece32064-bib-0113] Schmelzer, E. , W. Jahnen , and K. Hahlbrock . 1988 In situ localization of light‐induced chalcone synthase mRNA, chalcone synthase, and flavonoid end products in epidermal cells of parsley leaves. Proc. Natl Acad. Sci. USA 85:2989–2993. 16578833 10.1073/pnas.85.9.2989 PMC280128

[ece32064-bib-0114] Schmid, B. , F. A. Bazzaz , and J. Weiner . 1995 Size dependency of sexual reproduction and of clonal growth in 2 perennial plants. Can. J. Bot. 73:1831–1837.

[ece32064-bib-0115] Schulze, W. , and E.‐D. Schulze . 1995 The significance of assimilatory starch for growth in *Arabidopsis thaliana* wild‐type and starchless mutants Pp. 123–131 *in* Schulze E.‐D. and Caldwell M. M., eds. Ecophophysiology of photosynthesis. Springer‐Verlag, Berlin.

[ece32064-bib-0116] Searles, P. S. , S. D. Flint , and M. M. Caldwell . 2001 A meta analysis of plant field studies simulating stratospheric ozone depletion. Oecologia 127:1–10. 28547159 10.1007/s004420000592

[ece32064-bib-0117] Shugart, H. H. 1984 A theory on forest dynamics. The ecological implications of forest succession model. Springer‐Verlag, New York, NY, 278 pp.

[ece32064-bib-0118] Siipola, S. M. , T. Kotilainen , N. Sipari , L. O. Morales , A. V. Lindfors , T. M. Robson , et al. 2015 Epidermal UV‐A absorbance and whole‐leaf flavonoid composition in pea respond more to solar blue light than to solar UV radiation. Plant Cell Environ. 38:941–952. 25040832 10.1111/pce.12403

[ece32064-bib-0119] Smith, B. N. 2005 Photosynthesis, respiration, and growth Pp. 671–677 *in* Pessarakli M., ed. Handbook of Photosynthesis, 2nd edn Taylor & Francis Group, Boca Raton, FL.

[ece32064-bib-0120] Stafford, H. A. 1991 Flavonoid evolution – an enzymatic approach. Plant Physiol. 96:680–685. 16668242 10.1104/pp.96.3.680 PMC1080830

[ece32064-bib-0121] Suchar, V. A. , and R. Robberecht . 2015 Integration and scaling of UV‐B radiation effects on plants: from DNA to leaf. Ecol. Evol. 5:2544–2555. 26257869 10.1002/ece3.1332 PMC4523352

[ece32064-bib-0122] Sullivan, J. , and J. Rozema . 1999 UV‐B effects on terrestrial plant growth and photosynthesis Pp. 39–58 *in* Rozema J., ed. Stratospheric ozone depletion: the effects of enhanced UV‐B radiation on terrestrial ecosystems. Backhuys Publishers, Leiden, The Netherlands.

[ece32064-bib-0123] Systems, V. 2009 Vensim: Ventana Simulation Environment, 5.6 ed, http://www.vensim.com.

[ece32064-bib-0124] Tay, A. , A. Abdullah , M. Awang , and A. Furukawa . 2007 Midday depression of photosynthesis in *Enkleia malaccensis*, a woody climber in a tropical rainforest. Photosynthetica 45:189–193.

[ece32064-bib-0125] Taylor, R. M. , A. K. Tobin , and C. M. Bray . 1997 DNA damage and repair in plants Pp. 53–76 *in* Lumsden P. J., ed. Plants and UV‐B responses to environmental change. Cambridge Univ. Press, Cambridge, UK.

[ece32064-bib-0126] United Nations Environment Programme, E.E.A.P. 2012 Environmental effects of ozone depletion and its interactions with climate change: progress report, 2011. Photochem. Photobiol. Sci. 11:13–27. 22279621 10.1039/c1pp90033a

[ece32064-bib-0522] UVMRP (2010) UV‐B Monitoring and Research Program. http://uvb.nrel.colostate.edu/UVB/index.jsf. Accessed December 2010.

[ece32064-bib-0532] Ventana Systems , 2009 Vensim: Ventana Simulation Environment, 5.6 ed, http://www.vensim.com.

[ece32064-bib-0127] Wargent, J. J. , J. P. Moore , A. Roland Ennos , and N. D. Paul . 2009 Ultraviolet radiation as a limiting factor in leaf expansion and development. Photochem. Photobiol. 85:279–286. 18764892 10.1111/j.1751-1097.2008.00433.x

[ece32064-bib-0128] Warren, J. M. , J. H. Bassman , and S. Eigenbrode . 2002 Leaf chemical changes induced in *Populus trichocarpa* by enhanced UV‐B radiation and concomitant effects on herbivory by *Chrysomela scripta* (Coleoptera: Chrysomidae). Tree Physiol. 22:1137–1146. 12414373 10.1093/treephys/22.15-16.1137

[ece32064-bib-0129] Wayne, P. M. , and F. A. Bazzaz . 1993 Morning vs afternoon sun patches in experimental forest gaps: consequences of temporal incongruency of resources to birch regeneration. Oecologia 94:235–243. 28314037 10.1007/BF00341322

[ece32064-bib-0130] Weiner, J. 1995 On the practice of ecology. J. Ecol. 83:153–158.

[ece32064-bib-0131] Weiner, J. 2004 Allocation, plasticity and allometry in plants. Perspect. Plant Ecol. Evol. Syst. 6:207–215.

[ece32064-bib-0132] Winkel‐Shirley, B. 2002 Biosynthesis of flavonoids and effects of stress. Curr. Opin. Plant Biol. 5:218–223. 11960739 10.1016/s1369-5266(02)00256-x

[ece32064-bib-0133] Wright, I. J. , P. B. Reich , M. Westoby , D. D. Ackerly , Z. Baruch , F. Bongers , et al. 2004 The worldwide leaf economics spectrum. Nature 428:821–827. 15103368 10.1038/nature02403

[ece32064-bib-0134] Xu, D.‐Q. , and Y.‐K. Shen . 2005 External and internal factors responsible for midday depression of photosynthesis Pp. 287–297 *in* Pessarakli M., ed. Handbook of photosynthesis, 2nd edn Taylor & Francis Group, Boca Raton, FL.

[ece32064-bib-0135] Yamasaki, S. , N. Noguchi , and K. Mimaki . 2007 Continuous UV‐B irradiation induces morphological changes and the accumulation of polyphenolic compounds on the surface of cucumber cotyledons. J. Radiat. Res. 48:443–454. 17690531 10.1269/jrr.07046

[ece32064-bib-0136] Zhou, Y. H. , H. M. Lam , and J. H. Zhang . 2007 Inhibition of photosynthesis and energy dissipation induced by water and high light stresses in rice. J. Exp. Bot. 58:1207–1217. 17283375 10.1093/jxb/erl291

[ece32064-bib-0137] Ziska, L. H. , A. H. Teramura , and J. H. Sullivan . 1992 Physiological sensitivity of plants along an elevational gradient to UV‐B radiation. Am. J. Bot. 79:863–871.

